# Measuring heart rate variability using a heart rate monitor in horses (*Equus caballus*) during groundwork

**DOI:** 10.3389/fvets.2022.939534

**Published:** 2022-11-22

**Authors:** Chantal M. Kapteijn, Thibault Frippiat, Cees van Beckhoven, Hein A. van Lith, Nienke Endenburg, Eric Vermetten, T. Bas Rodenburg

**Affiliations:** ^1^Animals in Science and Society, Department of Population Health Sciences, Faculty of Veterinary Medicine, Utrecht University, Utrecht, Netherlands; ^2^Sportpaardenarts - Equine Sports Medicine, Laren, Netherlands; ^3^Heart for Horses, Oisterwijk, Netherlands; ^4^UMC Utrecht Brain Center, University Medical Center Utrecht, Utrecht, Netherlands; ^5^Department of Psychiatry, Leiden University Medical Center, Leiden, Netherlands; ^6^Adaptation Physiology Group, Wageningen University and Research, Wageningen, Netherlands

**Keywords:** heart rate, stress, equine-assisted intervention, novel object, equine, accuracy, exercise, RMSSD

## Abstract

Measuring physiological parameters of stress in horses during groundwork, for example when involved in equine-assisted interventions, is important to gain insight into the stress levels of the horses. Heart rate and heart rate variability can be used as physiological indicators of stress in horses. Heart rate monitors could be easily incorporated into practice, as they are not expensive and easy to use. However, it is questionable whether heart rate monitors present accurate heart rate variability results in exercising horses, similar to electrocardiograms. The aim of this study was to determine the accuracy of heart rate monitors for the assessment of heart rate variability in horses during groundwork exercise. Simultaneous telemetric electrocardiograms (Televet) and heart rate monitor (Polar H10 transmitter and M430 receiver, Hylofit electrodes) recordings were performed on 28 horses (15 mares and 13 geldings). Results indicate that the heart rate monitor accurately determined heart rate and time-domain heart rate variability parameters when compared to electrocardiograms during both baseline and groundwork conditions. As expected, heart rate significantly increased and the heart rate variability significantly decreased during groundwork compared to baseline conditions. This indicates that the heart rate monitor can be used to accurately determine heart rate variability during groundwork.

## Introduction

When evaluating stress and welfare in horses, a multidisciplinary approach considering both physiological and behavioral parameters is preferred (1, 2). Heart rate variability (HRV) is a physiological parameter commonly used to assess stress in farm animals including horses (3–5) and also in humans (6–9). Measurement of HRV is used to assess the regulation of the cardiovascular function by the autonomic nervous system (ANS) and is defined as the variation in the interbeat (RR) interval resulting from the balance between the sympathetic and parasympathetic activity at the sinus node of the heart (10). Generally, low HRV and decreased parasympathetic activity are associated with stress, exercise, excitement, or pathological conditions (11–13). HRV is a parameter that can be measured non-invasively in horses and, if it could be applied in practice easily, this could be a useful parameter for monitoring stress in horses.

Acquisition of RR intervals originating from a heart rate monitor (HRM) could make measurements of HRV more practically applicable compared to electrocardiogram (ECG) measurements. A HRM is cheap, easy-to-use and readily available (13), whereas an ECG is expensive and requires practitioners' experience and knowledge. However, the ECG is considered the gold standard to measure HRV, as they allow visual inspection of the depolarizations. There has been much controversy about best practices and whether HRM could give accurate HRV results based on *R*-waves. Horses have a pronounced *T*-wave which may be mistaken for an *R*-wave, therefore artifact correction of HRV originating from HRM data is needed (5). A review indicates that ECG is used in the majority of the studies that evaluate HRV, but a smaller number used a HRM (5). Ille et al. (14) validated a HRM with ECG for stationary horses. However, in non-stationary horses HRM measurements can differ significantly from ECG measurements (15–17). A more recent study by Frippiat et al. (18) demonstrated good agreement and concordance for HRV parameters between a HRM and an ECG in exercising horses. These differences in accuracy between studies in non-stationary horses may be explained by differences in the HRM used. In humans the HRM seems to produce accurate results during low-intensity exercise, when compared with ECG results, but accuracy decreases during high-intensity exercise (19–21). A recent study with Polar H10 in humans demonstrates excellent accuracy, also during high-intensity exercise (22). A quantitative review concludes that HRM demonstrate a small amount of error compared to ECG, but the authors considered this acceptable because of the improved practicality and compliance during HRV measurements (23). This suggests that more research is needed on the accuracy of HRM during different intensity activities and contexts in horses.

Accurately monitoring stress in horses through a practically applicable method, such as measuring HRV through HRM, could be valuable for example within the context of equine assisted interventions (EAI) (2, 3, 24, 25). EAI have become increasingly popular as a treatment option for humans with mental health issues (2, 3, 24, 26), but the effects of it on horses are not well-documented (2, 3, 24). EAI often include activities such as groundwork (24, 27) or an obstacle course (28) and some studies include exercises where the horses have to move through, under, or over an object (29). A study on horses performing backward movements, used an ECG to demonstrate a significant rise in HR and decrease in vagal tone compared to rest and forward movement, indicating stress since this was also correlated with behavioral parameters of stress (4). Other studies used HRM to investigate stress responses of horses to novel objects and demonstrated an increase in heart rate (HR), decrease in HRV and a correlation with behavioral parameters of stress (30–32). Furthermore, stress in horses may also lead to stress-related behaviors such as flight responses which may be dangerous to the human leading the horses during groundwork exercises. This indicates that scientific research on measuring the effects of groundwork is not only important for the welfare of the horse but also from a safety perspective. Although the accuracy of a HRM system has recently been demonstrated in exercising ridden horses (17), this has not been done for groundwork exercises including novel objects and this study did not include behavioral parameters of stress. The aim of the present study was to determine the accuracy of a HRM for the assessment of HRV in horses during groundwork exercises including novel objects, in order to determine whether this is a practically applicable method to monitor stress in these horses. It was hypothesized that HRV parameters obtained from HRM and ECG were not significantly different during exercise when corrected for artifacts. Furthermore, it was expected that groundwork exercises including novel objects would lower the HRV and would be correlated to behavioral parameters of stress.

## Materials and methods

### Subjects and procedure

In total, 29 sound horses and ponies from a riding school (Stal Groenendaal, Bunschoten, Netherlands) were included; 15 mares and 14 geldings. ECG measurement from 1 gelding could not be used due to bad recording and was therefore discarded from the dataset, leaving 28 horses (15 mares and 13 geldings) to be analyzed. This riding school accommodates mentally and physically disabled people to ride and interact with horses and also EAI for military veterans with post-traumatic stress disorder. The number of horses was calculated using the program G^*^Power (33). Average height was 154 ± 12 cm, average age was 10.6 ± 6.0 years. The horses were housed in individual boxes with straw bedding and ad libitum hay and were fed pellets once a day. Daily training of the horses consisted in being ridden for at least 1 hour. The horses were visually inspected and considered sound (no lameness and other obvious detectable causes of pain). A veterinarian assessed the ECG results to prevent inclusion of horses in the study that had arrhythmias.

Measurements with both ECG and HRM were simultaneously performed during baseline conditions (B) and groundwork conditions (GW) (before/after experimental design). Horses were given at least 5 minutes to adapt to wearing the elastic girth and equipment before baseline measurements started. However, the horses were already habituated to wearing girths previously as they were being ridden regularly. Baseline recordings in the stable lasted for 5 minutes and were performed in the horses' own stables while standing still and being haltered. Food was removed 30 minutes prior to the start of the baseline recording. Horses were taken to the groundwork exercise with novel objects within 10 minutes after the baseline recordings. Recordings during groundwork were at least 2 minutes long. Most horses took more than 2 minutes to complete the groundwork exercise, but the horses that took <2 minutes were led through another time to gain at least 2 minutes of measurements.

**Figure 1:**
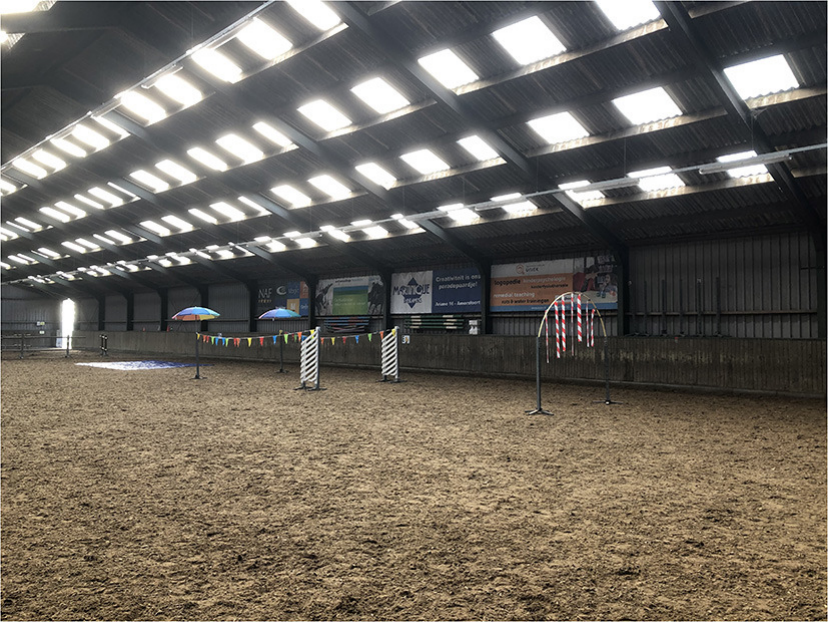
This picture presents an overview of groundwork exercise consisting of 3 novel objects, starting with the arch with ribbons on the right hand side and including a row of flag and umbrellas and ending with a plastic floor cover, used for the horses (*n* = 28) (^©^xQ. Nijenhuis).

The horses were led by hand by 8 different handlers, the weight of the handlers was 72.8 ± 13.9 kilogram and age was 35.0 ± 18.7 years. All handlers were experienced horse handlers (> 1 year of experience) that were either students of veterinary medicine or were involved in training (police) horses. All handlers were instructed to follow a standardized procedure. Objects could be approached maximally 5 minutes and if the horse did not pass it by that time the horse would be led around it. Minimal pressure was applied to the horses while being led on a halter using a lead rope and no other specific tools or punishments were used. The novel objects (see Figure 1) were placed in an inside arena at the riding school, about 4 meter of the outside walls. The horses were habituated to the inside arena, although they were not familiar with the objects. The objects consisted of three elements that were placed 8 meter apart: the horse had to walk under an arch with ribbons, in between a row of flags and umbrellas and over a plastic floor cover. The horses were led with a halter and rope, in walking pace keeping them in low intensity exercise throughout the measurements.

### Heart rate and heart rate variability

**Figure 2:**
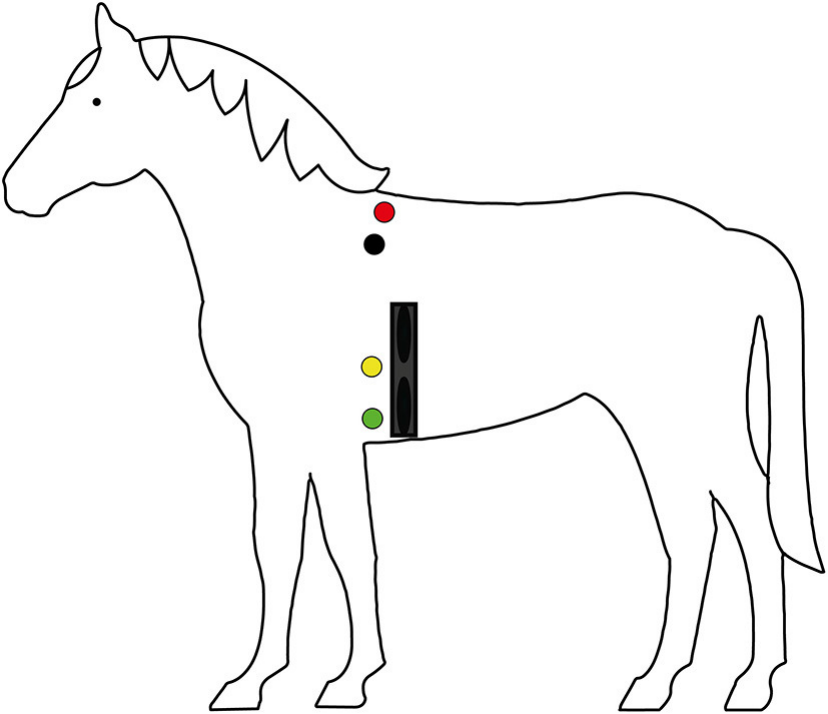
Graphic representation of placement of the Televet electrode (red, black, yellow, and green dots) and Polar Hylofit (dark blue band) on the horses (*n* = 28). The red electrode is attached dorsally to the right side of the horse. The black, yellow and green electrodes were attached to the left side of the horse.

The Hylofit electrodes (Equinics, Tølløse, Denmark), Polar H10 Heart Rate Sensor transmitter and Polar M430 receiver (Polar Electro Nederland, Utrecht, Netherlands) were used to obtain interbeat interval (IBI) recordings. The Hylofit, containing 2 electrodes, was attached with a Velcro elastic girth after applying Aquasonic 100 conducting gel, location is shown in Figure 2. Kruuse ECG Electrodes were connected to the Televet 100 (Engel Engineering, Heusenstamm, Germany) which was also attached to the girth to prevent movement of the equipment while the horse is walking, electrode location is shown in Figure 2.

The Televet data were manually corrected using Televet software version 6.0.0 before RR-intervals being exported. All raw IBI were then imported into the software (Kubios HRV, University of Eastern Finland, Kuopio Finland) for HRV parameter analysis after applying the strong correction filter (34). The following HRV parameters were used: time-domain parameters including standard deviation of the successive R–R intervals (SDNN) and root mean square of the successive differences (RMSSD) (5, 10). These time-domain parameters are more easy to understand compared to frequency-domain parameters and therefore more often reported in literature (13).

### Behavior recordings

Oppo A5 2020 phones were used to videorecord the horses and these video recordings were used for behavior analysis. A comprehensive ethogram (Table 1) was used to analyze stress-related behavior in the horses. Snorting is defined as a forceful quick exhalation and is associated in literature with olfactory investigation, posturing and aggression (40, 41), and fear/anxiety (42). Whites of the eyes is defined as the sclera being exposed, which is associated with pain (42) and stress (35, 43). Flight response is defined as moving away from an object in an accelerated locomotion, which is an adaptive response for a prey species when faced with external stressors (44). Behaviors were scored from the videos using The Observer XT (15.0 Noldus, Wageningen, Netherlands). Behaviors that did not occur or rarely occurred (rearing, bucking, head shaking, pawing, defecating, urinating, screaming, yawning, flehmen response) were excluded from the analysis. Throughout the study, one observer performed the observations and was trained by practicing with this specific ethogram beforehand. Reliability analysis (Cohen's kappa) showed that intra-observer reliability was 91.2% for duration and 86.2% for frequency.

**Table 1:** Ethogram of the included horse behaviors.

**Behavior**	**Definition**
Lip licking	The horse opens the mouth, thereby shortly extending and retracting the tongue (including licking the lips) (35–37).
Chomping	The horse makes chewing movements with the jaw (without the tongue being visible), may include teeth grinding, not directed to food and/or other objects (35–37).
Tail swishing	The horse moves the tail horizontally or vertically, and repeatedly swishes it from one side to the other of its quarters or up and down (36, 38, 39).
Vocalization	The horse neighs (low soft sound) or whinnies (starts high pitched and may end in neigh), and vocalizes in another way (excluding snort) (36, 37).
Snorting	Low-pitched sound as a consequence of air being exhaled through nostrils (37, 38, 40–42).
Eye white	The horse is showing eye white around the pupil (35, 42, 43).
Flight response	The horse moves away suddenly/rapidly, usually in response to an external stimulus in accelerated locomotion (44, 45).
Fear response	The horse shows a sudden movement or muscle contractions in response to an external stimulus (42, 45).
Head up	The horse throws its head up in accelerated movement with the nose reaching above the back line (4, 36, 45).

### Statistical analysis

Statistics were performed using SPSS statistics (version 26, IBM Corp., Armonk, NY, USA). Two-sided probabilities were estimated throughout. The exact Kolmogorov-Smirnov one-sample test was used to check Gaussianity of the data and showed that for the HR, SDNN, and RMSSD, the differences between the two compared methods were normally distributed. A one-sample *t*-test was performed to see whether they differed significantly from the expected null hypothesis (there is no difference between the two; difference = 0, *P*-value of < 0.05 was considered significant). Since the *P*-values of the one-sample *t*-test were not significant, Bland-Altmann plots were constructed in which the differences were plotted against the mean and were then visually inspected. A linear regression was applied to detect proportional bias within the datapoints of the Bland-Altmann plot. Differences in HRM data between the baseline and groundwork were assessed with paired Student's *t*-tests for normally distributed parameters (HR and HRV) and α = 0.05. Statistical significance represented by *P*-values may not necessarily confirm practical importance. In our opinion, the size of the observed effects is perhaps more important than statistical significance. Therefore, besides *P*-values, estimated effect sizes were calculated and reported. To estimate the relative magnitude of the normally distributed differences, Cohen's *d* effect size coefficients were calculated, i.e., the difference between the two means divided by the pooled SD. A commonly used interpretation—based on benchmarks suggested by Cohen (46)—is to refer to effect sizes as: zero or nearly zero effect, 0 ≤ |*d*| < 0.2; small effect, 0.2 ≤ |*d*| < 0.5; moderate effect, 0.5 ≤ |*d*| < 0.8; and large effect, |*d*| ≥ 0.8. Effect sizes were considered meaningful if |*d*| ≥ 0.5. With the number of horses used in this study (*n* = 28), a two-tailed one-sample Student's *t*-test, a threshold of significance = 0.05, and a power of ≈0.80, we were able to detect an effect size |*d*| of 0.55 or more (47).

Between the different parameters Pearson's linear correlation coefficients (*r*, normally distributed) or Spearman coefficients of rank correlation (*R*^*S*^, not normally distributed) were calculated. The significance (*P* < 0.05) was assessed by a two-tailed test based on the *t* statistic. Although there are no hard and fast rules for describing correlational strength, the following guidelines are widely accepted: weak correlation, 0 ≤ |*r* or *R*^*S*^| < 0.3; moderate correlation, 0.3 ≤ |*r* or *R*^*S*^| < 0.7; and strong correlation, 0.7 ≤ |*r* or *R*^*S*^| ≤ 1.0.

## Results

The ECG measurement from 1 gelding could not be used due to bad recording and was therefore discarded from the dataset, leaving 28 horses to be analyzed. Both systems were tolerated well by the horses and no interference between the systems was observed. Recordings during groundwork were at least 2 minutes long and lasted on average 6.3 ± 3.3 minutes.

### Accuracy mean heart rate (HR)

**Figure 3:**
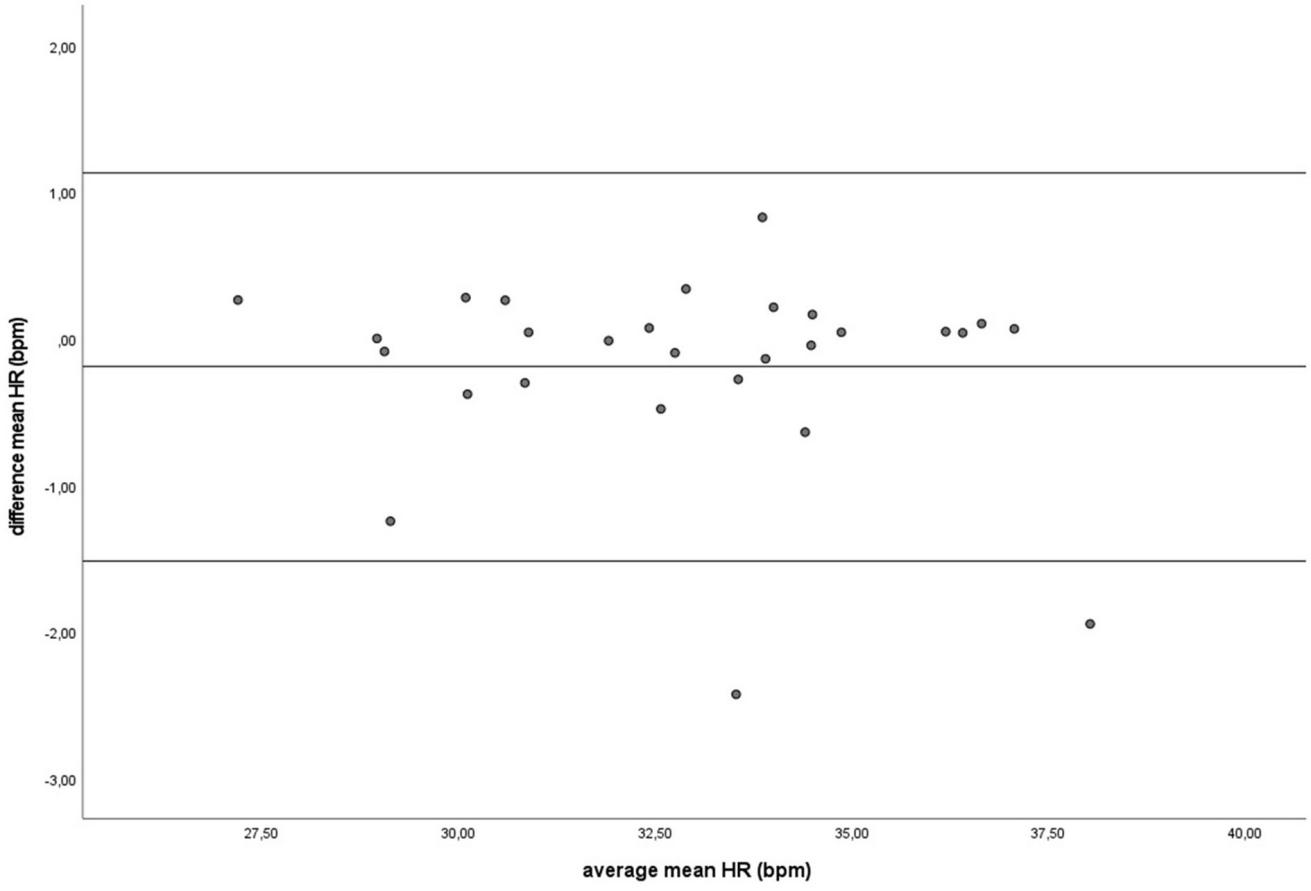
The Bland-Altman plot for the mean heart rate in beats per minute during baseline measurement, with the difference between sampling methods (*y*-axis) plotted against the mean of the two methods (*x*-axis). Each data point represents an individual horse (*n* = 28). The solid lines represent the upper and lower limit of the 95% confidence interval, respectively and the middle line represents the mean difference.

**Figure 4:**
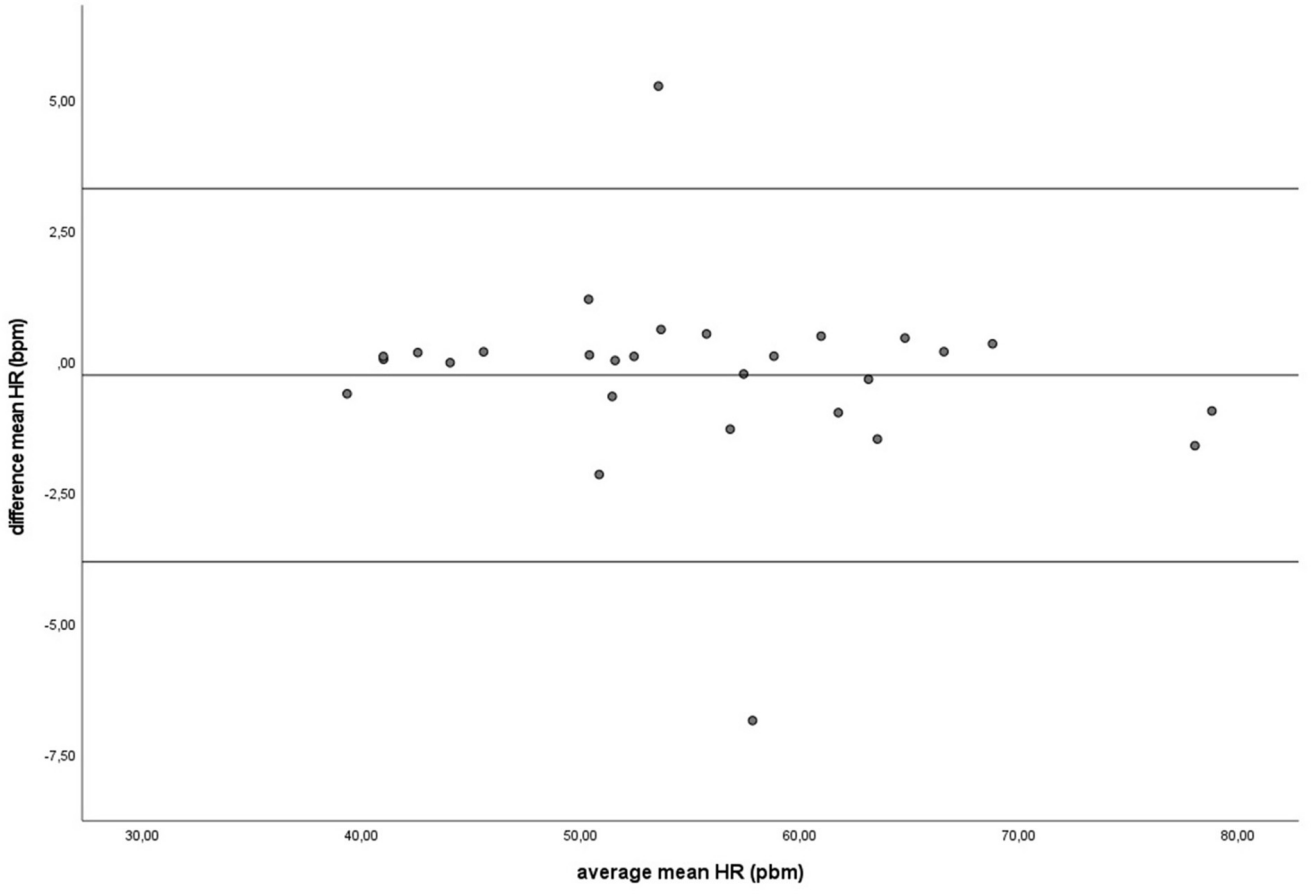
The Bland-Altman plot for the mean heart rate in beats per minute during groundwork, with the difference between sampling methods (*y*-axis) plotted against the mean of the two methods (*x*-axis). Each data point represents an individual horse (*n* = 28). The solid lines indicate the upper and lower limit of the 95% confidence interval, respectively and the middle line represents the mean difference.

During baseline measurements (B) and groundwork (GW), the results indicated that both recording methods did not differ significantly for mean HR and had zero or nearly zero effect (B: *df* = 27, *t* = −1.493, *P* = 0.147, |*d*| = 0.069, GW: *df* = 27, *t* = −0.816, *P* = 0.421, |*d*| = 0.027). During B, the mean of the difference between HRM and ECG was −0.2 ± 0.7 bpm, with 25 of the 27 recordings (93%) within the 95% confidence interval (see Figure 3). During GW, the mean of the difference between HRM and ECG was −0.3 ± 1.8 bpm, with again 25 of the 27 recordings (93%) within the 95% confidence interval (see Figure 4). A linear regression showed no proportional bias for the mean HR during B (*df* = 27, β = 0.033, *P* = 0.496) or GW (*df* = 27, β = 0.029, *P* = 0.403).

### Accuracy of time based HRV parameters (SDNN, RMSSD)

**Figure 5:**
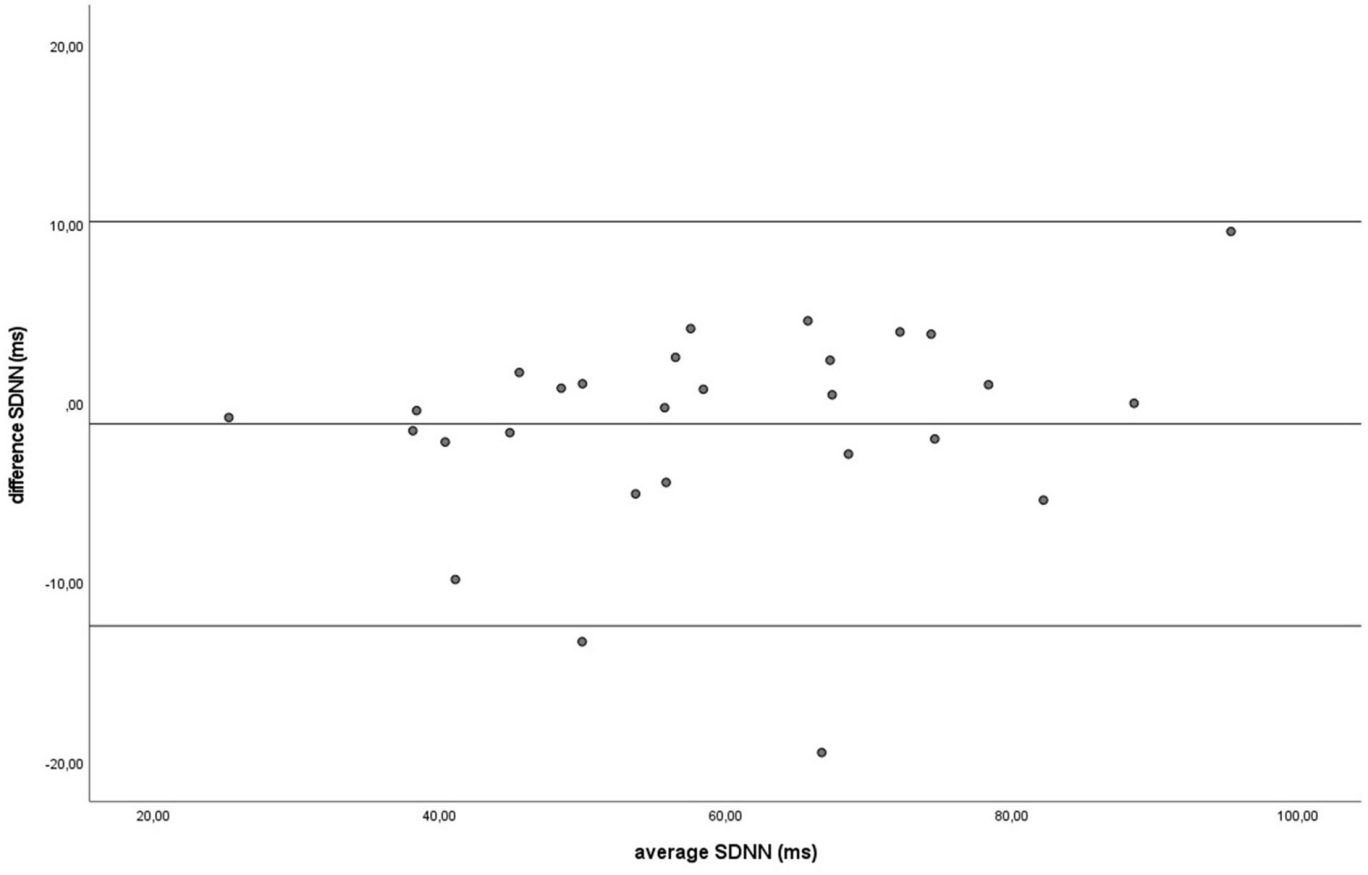
The Bland-Altman plot for the SDNN in milliseconds during the baseline, with the difference between sampling methods (*y*-axis) plotted against the mean of the two methods (*x*-axis). Each data point represents an individual horse (*n* = 28). The solid lines indicate the upper and lower limit of the 95% confidence interval, respectively and the middle line represents the mean difference.

**Figure 6:**
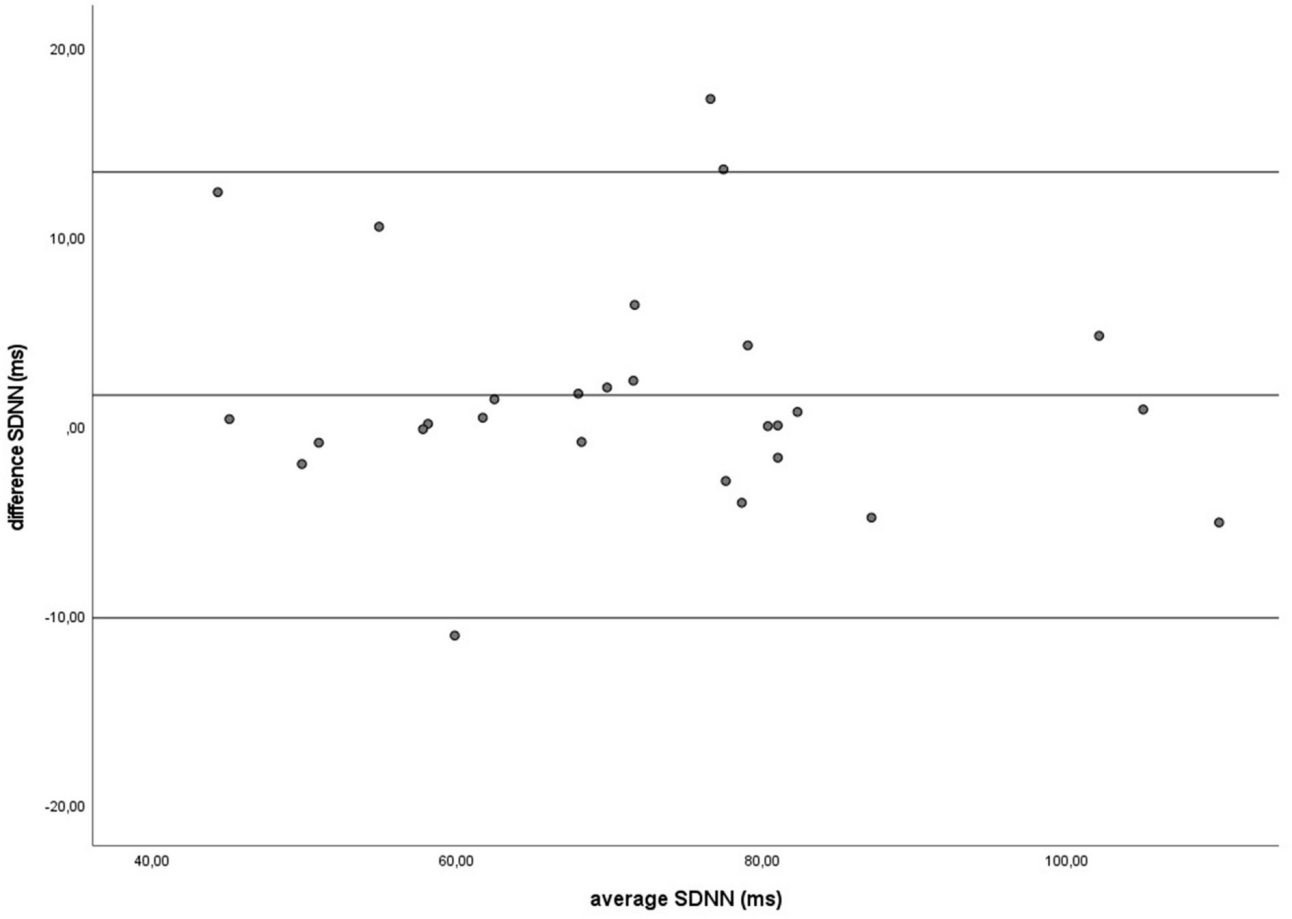
The Bland-Altman plot for the SDNN in milliseconds during the groundwork, with the difference between sampling methods (*y*-axis) plotted against the mean of the two methods (*x*-axis). Each data point represents an individual horse (*n* = 28). The solid lines indicate the upper and lower limit of the 95% confidence interval, respectively and the middle line represents the mean difference.

**Figure 7:**
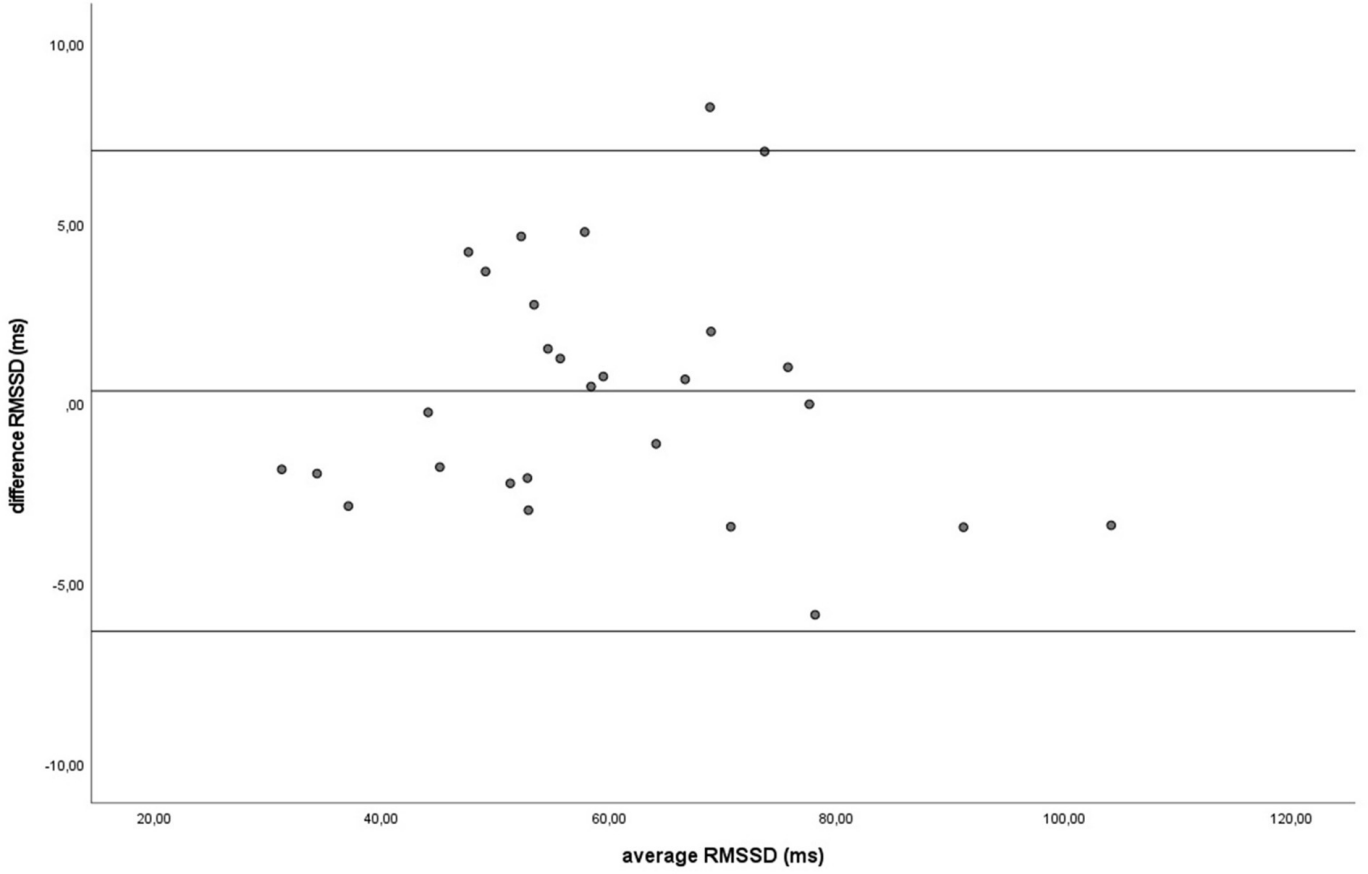
The Bland-Altman plot for the RMSSD in milliseconds during the baseline, with the difference between sampling methods (*y*-axis) plotted against the mean of the two methods (*x*-axis). Each data point represents an individual horse (*n* = 28). The solid lines indicate the upper and lower limit of the 95% confidence interval, respectively and the middle line represents the mean difference.

**Figure 8:**
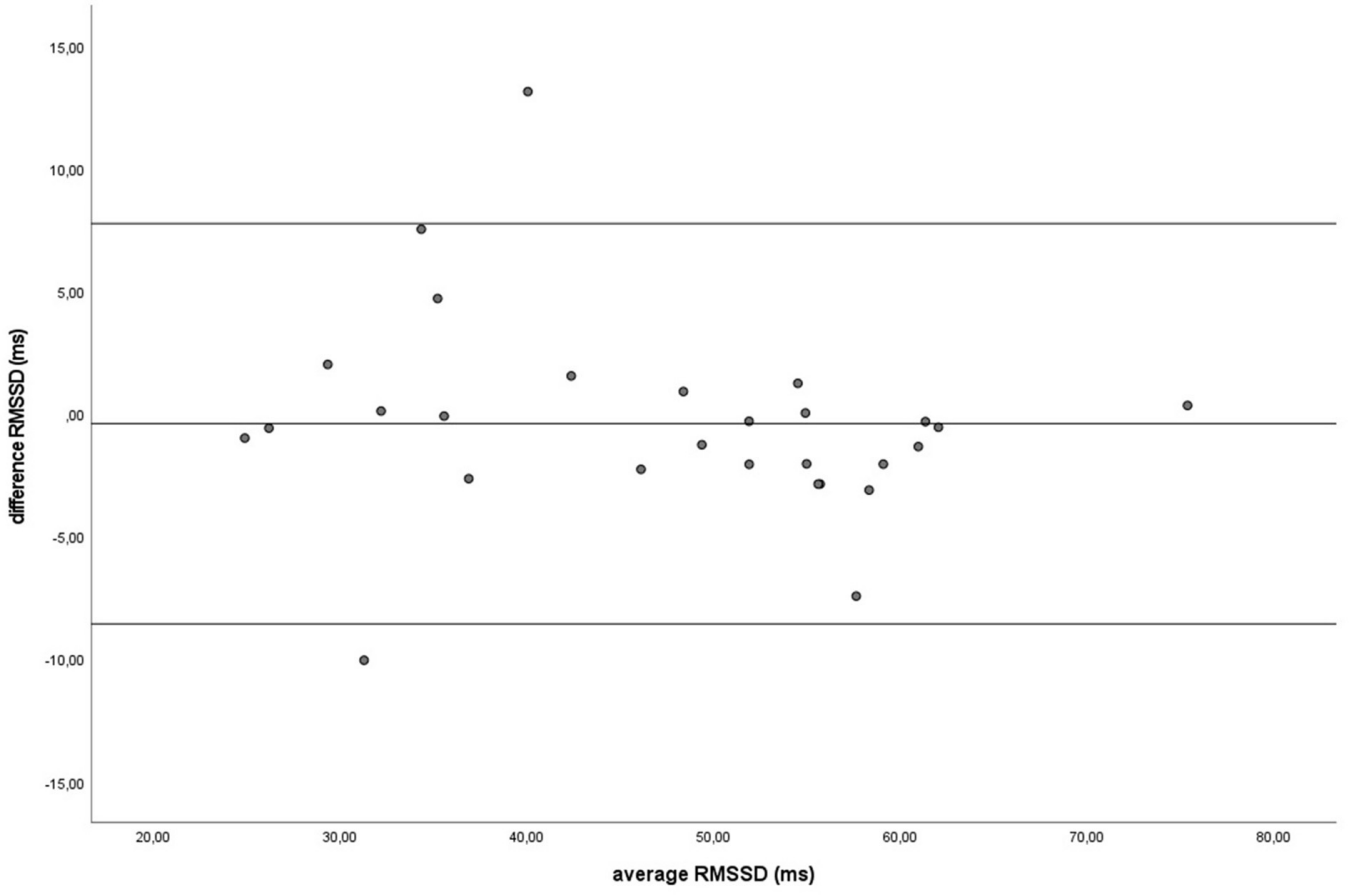
The Bland-Altman plot for the RMSSD in milliseconds during groundwork, with the difference between sampling methods (*y*-axis) plotted against the mean of the two methods (*x*-axis). Each data point represents an individual horse (*n* = 28). The solid lines indicate the upper and lower limit of the 95% confidence interval, respectively and the middle line represents the mean difference.

Similar to the HR data, the recording methods also did not differ significantly and had a zero or nearly zero effect respectively for the SDNN (B: *df* = 27, *t* = −1.062, *P* = 0.297, |*d*| = 0.068, GW: *df* = 27, *t* = 1.405, *P* = 0.171, |*d*| = 0.093) and RMSSD (B: *df* = 27, *t* = 0.531, *P* = 0.600, |*d*| = 0.021, GW: *df* = 27, *t* = 0.524, *P* = 0.605, |*d*| = 0.032). During B, the SDNN mean of the difference between HRM and ECG was −1.2 ± 5.8 ms, with 25 of the 27 recordings (93%) within the 95% confidence interval (see Figure 5). During GW, the SDNN mean of the difference between HRM and ECG was 1.6 ± 6.0 ms, with 25 of the 27 recordings (93%) within the 95% confidence interval (see Figure 6). During B, the RMSSD mean of the difference between HRM and ECG was 0.3 ± 3.4 ms, with 26 of the 27 recordings (96%) within the 95% confidence interval (see Figure 7). During GW, the RMSSD mean of the difference between HRM and ECG was −0.4 ± 4.2 ms, with 25 of the 27 recordings (93%) within the 95% confidence interval (see Figure 8). A linear regression showed no proportional bias for the SDNN (B: *df* = 27, β = −0.084, *P* = 0.215; GW: *df* = 27, β = −0.042, *P* = 0.555) and the RMSSD (B: *df* = 27, β = −0.015, *P* = 0.706; GW: *df* = 27, β = −0.065, *P* = 0.307). This means that measurements of the HRM system, both during baseline measurements and during groundwork are within the limits of agreement for both HR and time domain HRV parameters.

For an overview of the results on both HR and SDNN and RMSSD (see Table 2).

**Table 2:** Table representing the mean difference between the ECG and HRM and the standard deviation of the differences and the upper and lower limit of the 95% confidence interval, for the mean heart rate (HR) in beats per minute, and time-based parameters of heart rate variability (SDNN in ms and RMSSD in ms) during both the baseline conditions (B) and groundwork (GW) for the horses (*n* = 28).

	**Mean HR B**	**Mean HR GW**	**SDNN B**	**SDNN GW**	**RMSSD B**	**RMSSD GW**
Average ± SD	−0.19 ± 0.68	−0.28 ± 1.82	−1.16 ± 5.75	1.6 ± 6.01	0.34 ± 3.41	−0.41 ± 4.16
Upper limit	1.13	3.28	10.12	13.39	7.02	7.75
Lower limit	−1.52	−3.48	−12.43	−10.19	−6.34	−8.58

### Comparison of baseline conditions to groundwork

A paired *t*-test showed that the mean HR across horses was significantly higher with a large effect size (*df* = 27, *t* = −11.729, *P* < 0.001; |*d*| = 3.036) during GW (55.6 ± 10.2 bpm) compared to B (32.8 ± 2.7 bpm). The SDNN also was significantly higher with a moderate effect size (*df* = 27, *t* = −3.493, *P* = 0.002; |*d*| = 0.748) during GW (72.7 ± 16.8 ms) compared to B (58.8 ± 17.6 ms). The RMSSD was significantly lower with a large effect size (*df* = 27, *t* = 3.892, *P* = 0.001; |*d*| = 0.841) during GW (47.2 ± 12.7 ms) compared to B (60.0 ± 16.5 ms).

### Correlation HRV and behavior

A moderate positive correlation was found between mean HR and whites of the eyes shown (*df* = 27, *r* = 0.486, *P* = 0.009) and a tendency was found for snorting (*df* = 27, *R*^*S*^ = 0.369, *P* = 0.054). A moderate positive correlation between the SDNN and the flight response (*df* = 27, *R*^*S*^ = 0.419, *P* = 0.026) and a moderate negative correlation between the RMSSD and snorting (*df* = 27, *R*^*S*^ = 0.376, *P* = 0.049) was found. No correlations were found for lip licking, chomping, tail swishing, vocalizations, fear responses or head up and HR or HRV parameters.

## Discussion

The aim of this study was to determine whether the HRM can determine HRV accurately in horses during groundwork. The results from HRM and ECG systems do not differ significantly and had a zero or nearly zero effect size for HR and HRV parameters such as SDNN and RMSSD, indicating that the HRM provides accurate results. During groundwork, the HR and SDNN increased and RMSSD decreased as expected, due to increased arousal when passing novel objects. These results confirm a recent study that showed that HRM in exercising horses provide accurate data (18). In our study data were processed in Kubios and applying the strong correction factor gave the best result. This is in line with the study of van Vollenhoven et al. (34) on the reliability and repeatability of an HRM, indicating that a HRM can be used during movement, but may require the use of a correction factor. The fact that previous studies on exercising horses showed significant differences between HRM and ECG (15–17) can be explained by improved technologies, such as electrodes used. During groundwork, HR and SDNN were significantly higher and the RMSSD was significantly lower compared to baseline conditions as expected, with a large effect size for HR and RMSSD and a moderate effect size for SDNN. These results demonstrated that HRM do reflect expected differences in HRV due to differences in arousal when passing novel objects during GW compared to the B.

Our results on increased HR and decreased RMSSD in horses during groundwork are in line with other studies that used novel objects in an arena (30) or that measured HRV parameters during riding (48), but also during road transport of horses (49). Our results on an increased SDNN during groundwork are in agreement with some studies that demonstrated increased SDNN during transport (49) and transrectal palpation (50), but in contradiction to other studies on backward walking (4), restraint (51) or novel objects (30). The results indicate increased autonomic regulation, and a decrease in parasympathetic activity within the autonomic regulation. The SDNN reflects long term variability of cardiac activity and activation of the autonomic nervous system, both the sympathetic and parasympathetic activity (9, 52). The RMSSD reflects the short-term HRV and parasympathetic activity (9, 11, 52). The increased SDNN can be attributed to an increase in sympathetic activity that outweighs the decrease in parasympathetic activity. Generally, low parasympathetic activity is associated with stress (11, 12). Furthermore, a positive correlation between HR and showing whites of the eyes, and a negative correlation between RMSSD and snorting was found. This is in line with Safryghin et al. (42), who found that physiological (HR) and behavioral responses to a novel object were linked, with individuals having a higher HR also displaying more stress-related behaviors. Furthermore, Rietmann et al. (4) showed an increase in HR and decreased parasympathetic activity as a result of enforced backward movement which was correlated with behavioral parameters of stress, compared to forward movement. The current study also showed a moderate positive correlation between the SDNN and the flight response.

An important limitation in our study is that baseline measurements were performed with the horse standing still and did not control for movement. This means that no distinction can be made between the effects of physical exercise and psychological stress. Another limitation of this study is that the SDNN can be influenced by the duration of the measurement, as an increased time period automatically leads to increased SDNN (5). The increased SDNN in our study may (partly) be explained by the duration, since baseline measurements were generally shorter compared to groundwork measurements. Future research should investigate if the effects of groundwork with novel objects on HRV parameters are caused by physical exercise or psychological stress. This can be done for example to include a control during which the horses are exercised in walking pace in a comparable inside arena without novel objects before performing the groundwork exercise. Furthermore, the length of the measurements should remain the same to be able to compare baseline, control and groundwork conditions.

## Conclusion

In conclusion, the HRM system as described in this study accurately measures time-based parameters of HRV in horses during groundwork, after applying the strong correction factor in Kubios software. Future research should investigate if the changes observed in the current study between rest and groundwork exercise are induced by physical exercise and/or psychological stress from novel objects in horses.

## Data availability statement

The raw data supporting the conclusions of this article will be made available by the authors, without undue reservation.

## Ethics statement

Ethical review and approval was not required for the animal study because the study set up fell outside the category of animal experiments that require licensing according to the Dutch law. The Animal Welfare Body did an internal ethical review and concluded that the study did not warrant a license under the Dutch law, and it was approved by them. No invasive procedures were performed and the horses performed activities that were part of their normal training routines at the stable. Furthermore, these horses are accustomed to perform activities with a variety of (unfamiliar) people and were never forced through an object and were allowed to go around them if needed. These horses are privately owned and their owners gave their written informed consent to participate to the study.

## Author contributions

CK writing the article, conception/design of the work, data analysis, and acquisition and interpretation. TF data analysis and interpretation and revising the work. CB data acquisition and interpretation. HL data analysis and revising the work. NE and EV conception and design of the work and revising the work. TR conception and design of the work, data interpretation, and revising the work. All authors contributed to the article and approved the submitted version.

## Funding

This research was funded by the Dr. C. J. Vaillantfonds, K. F. Hein Fonds, Karel Doormanfonds and one funder that wants to remain anonymous.

## Conflict of interest

The authors declare that the research was conducted in the absence of any commercial or financial relationships that could be construed as a potential conflict of interest.

## Publisher's note

All claims expressed in this article are solely those of the authors and do not necessarily represent those of their affiliated organizations, or those of the publisher, the editors and the reviewers. Any product that may be evaluated in this article, or claim that may be made by its manufacturer, is not guaranteed or endorsed by the publisher.
